# Healthy aging in rats is associated with a decline in the ability to inhibit maladaptive responses, but not in measures of self-control by delayed gratification

**DOI:** 10.3389/fnagi.2025.1579934

**Published:** 2025-05-30

**Authors:** Adam T. Brockett, Xavier Sciarillo, Xuan Li, Matthew R. Roesch

**Affiliations:** ^1^Department of Psychology, University of Maryland, College Park, MD, United States; ^2^Program in Neuroscience and Cognitive Science, University of Maryland, College Park, MD, United States; ^3^Department of Biological Sciences, University of New Hampshire, Durham, NH, United States

**Keywords:** aging, cognitive health, cognitive control, reward, cognition

## Abstract

**Introduction:**

While it is often assumed that aging is associated with a general decline in cognitive health and decision-making, behavioral and neural evidence suggests that this decline may not be as broad as once thought. Cognitive health can be measured in various ways but is often subdivided into our ability to adapt motor plans to rapidly changing sensory information (inhibitory control) as well as our ability to make effectively delay gratification (self-control).

**Methods:**

To examine how aging impacts these aspects of cognitive health across the lifespan, we tested rats of various ages on the stop-change task, a measure of inhibitory control, and reset and no-reset versions of the diminishing returns task, a measure of self-control by delayed gratification.

**Results:**

In Experiment 1, we show that 10–12-month-old rats performed fewer trials compared to rats 3–4 months of age and exhibited significant differences in some measures of inhibitory, but not self, control as measured by diminishing returns. In Experiment 2, we show that 21–23-month-old rats show significant deficits in multiple measures of inhibitory control but largely resemble 14–15-month-old rats on measures of self-control. The results from both experiments highlight that aged rats tend to be less sensitive to delays in reward. Finally, we show that overexpression of an epigenetic enzyme (histone deacetylase 5)—thought to be elevated in aged individuals—worsens inhibitory control.

**Conclusion:**

Across these experiments we show that the impact of aging on cognitive health is not unitary, in that aging negatively impacts the adaptation of motor actions independent of self-control.

## Introduction

The UN World Population Prospects (2022 edition) recently estimated that by the year 2100, 25% of the world’s population will be aged 65 or older (UN, 2022). The same metric estimates that this demographic currently stands at approximately 10%, suggesting a rapid rise in the population of older individuals over the next 75 years (UN, 2022). In order to ensure a greater quality of life for aging individuals in addition to protecting the financial stability of the global economy, a greater understanding of the neurobiological changes that accompany healthy aging is needed.

While conventional wisdom often posits that little good comes with advanced age, research investigating the behavioral and neurobiological changes associated with aging has presented a more nuanced perspective (Buckner, 2004; Samanez-Larkin and Knutson, 2015). While cognitive health does decline with age, the deficits appear to break down along measures of fluid and crystallized intelligence, with fluid intelligence being the most negatively impacted (Samanez-Larkin and Knutson, 2015). Fluid intelligence comprises a suite of problem solving abilities that often work in concert with control processes, such as the ability to adapt motor plans in the face of changing sensory information, inhibitory control as well as the ability to reason about the optimal allocation of resources, value-based decision making (Braver and Barch, 2002; Manard et al., 2014; Yee et al., 2019). Research in humans suggests that deficits in inhibitory control tend to grow with age, but that similarly aged individuals maintain the ability to make value-guided decisions compared to younger individuals (Manard et al., 2014; Samanez-Larkin and Knutson, 2015; Yee et al., 2019). Still, uncertainty remains given that many of these assessments are made across studies, tasks, and individuals.

Rodent models offer a more tractable model to study aging, as the average lifespan of a rat is approximately 2 years (Gorbunova et al., 2008; Mitchell et al., 2015). Despite this advantage, many rodent aging studies treat age as a binary variable (i.e., young vs. aged), imposing arbitrary distinctions between stages of the lifespan. This decision can limit research’s ability to study the onset of changes in cognitive health with age. To address this, we assessed the ability of two cohorts of rats to perform the stop-change task, a measure of inhibitory control, and the diminishing returns task, a measure of self-control by delayed gratification across two experiments. The first experiment examined performance on both tasks between rats aged 3–4 and 10–12 months of age. The second assessed the same cohort of rats on the same measures at 14–15 and 21–23 months of age. We found in the first experiment that deficits in inhibitory control but not self-control started to vary as a function of age, and that in experiment two 21–23-month-old rats showed clearer evidence for declines in inhibitory control but performed similarly to 14–15-month-old rats in terms of self-control. Finally, we took advantage of the tractability of rodent models to examine the impact of epigenetic modification on age related processes. The accumulation of epigenetic modifications over the course of the lifespan has been associated with age-related declines in cognitive abilities (Fischer et al., 2010; McIntyre et al., 2019). Here we selectively upregulated histone deacetylase 5 (HDAC5) activity in anterior cingulate cortex (ACC), a protein previously implicated in inflexible decision-making to test whether this could contribute to age-related declines in cognitive processing across both tasks (Pribut et al., 2021). HDAC5 has been implicated in memory loss (Agis-Balboa et al., 2013) and musculoskeletal health (Walsh and Van Remmen, 2016) with age, but its role in cognition and its impact of circuit level brain functioning has been incompletely studied. Some have suggested that HDAC inhibitors generally, may lessen age-related cognitive decline, but the role of HDACs in maintenance of cognitive ability with age remains an open question (McIntyre et al., 2019). Here, we examined if we can mimic loss of cognitive function seen with aging by overexpressing HDAC in a brain area (ACC) that we know ACC is necessary for adapting motor plans during tests of cognitive control (Brockett et al., 2020; Bryden et al., 2019) and reward-guided decision-making involving delays to reward (Vázquez et al., 2024a; Vázquez et al., 2024b). Further, given our previous work suggesting HDAC5 overexpression promotes inflexible decision making, we chose to specifically overexpress HDAC5, a class II HDAC that shuttles between the nucleus and cytoplasm in response to intracellular signaling and is highly expressed in cortical regions (Kim et al., 2012; McQuown and Wood, 2011; Yang and Grégoire, 2005). Consistent with correlations between HDAC5 activity and cognitive decline observed with age, overexpression worsened measures of motor impulsivity. Collectively, these findings support the emerging human literature suggesting that age-related changes in control processes are not unitary, and that inhibitory control processes are distinct from self-control processes.

## Materials and methods

### Animals

Four female and five male Sprague Dawley rats (*n* = 9) were obtained at approximately 2–3 months of age from Charles River Laboratories. A second cohort of 6 female and 6 male Sprague Dawley rats (*n* = 12) were obtained at 9–11 months of age from Charles River Laboratories. The 9–11-month-old cohort were listed as former breeders. No other information about breeding history or other past experiences was provided. Both cohorts of rats were housed on a 12/12 h light/dark schedule with lights on at 6:00 am EST. All training and behavioral testing occurred between 10:00 am and 6:00 pm EST. Food was provided *ad libitum*, but rats were water restricted throughout training and testing. Water was provided for approximately 20 min each day of training/testing after rats had completed the day’s session. All experimental procedures were approved by the University of Maryland Animal Care and Use Committee and conformed to the guidelines set forth by the National Research Council (National Research Council (US) Committee for the Update of the Guide for the Care and Use of Laboratory Animals, 2011).

### Experimental timeline

**FIGURE 1:**
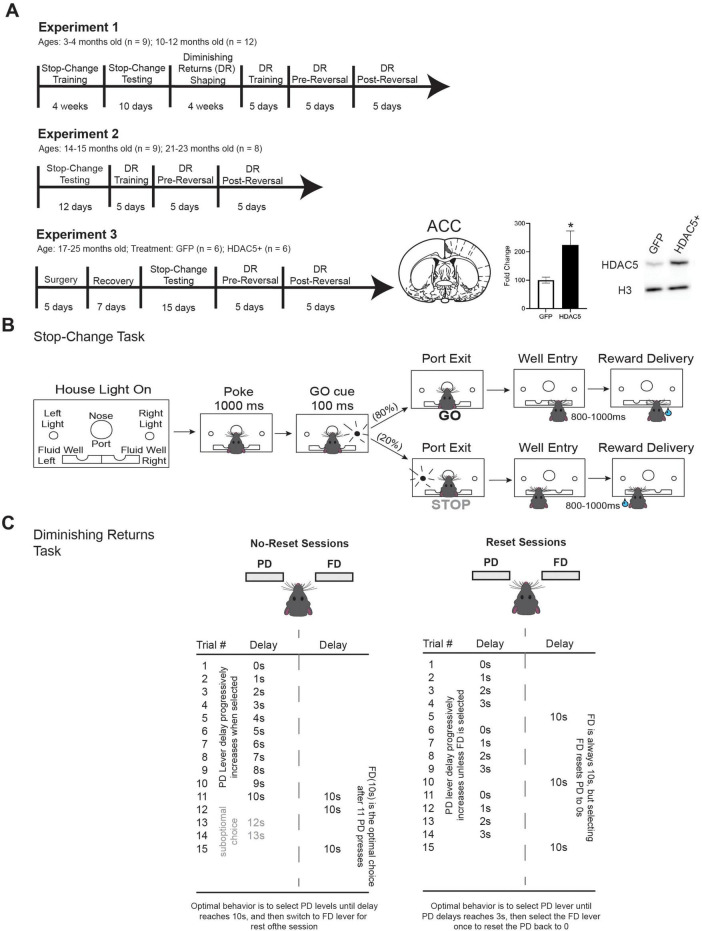
Timeline and task design. **(A)** Experimental timeline. In Experiment 1, Rats were trained on the stop-change task and then tested for 10 days. Rats were then trained and tested on the diminishing returns task for an additional 10 days. In Experiment 2, the same rats were tested 11 months later both the stop-change task and diminishing returns task. In Experiment 3, rats from cohort 1 and 2 were combined into a single cohort, and the rats, now 17–25-months of age (*n* = 12), underwent bilateral virus injection surgery to target neurons in ACC with either GFP (control) or to increase HDAC5 expression (HDAC5 +). The fold increase in HDAC5 mRNA expression is displayed along with a schematic showing ACC and an example immunoblot image. *Indicated *p* < 0.05. **(B)** Schematic for stop-change task. Following the house lights rats made a nose poke for 200 ms before a light cue was illuminated on either the right or left side. On 80% of trials (GO trials) this light corresponded to the correct direction that rat needed to move to receive reward. On 20% of the trials a second light was illuminated after the initial GO cue directing the rat to inhibit their initial response to the first cue in favor of making a response in the direction of the second cue. **(C)** Schematic for the diminishing returns task. In the diminishing returns task, rats choose between two troughs. One delivers a reward after a fixed delay (FD), while the other delivers reward on a progressive delay (PD) schedule where the delay increases by 1 s with each entry into the trough. In the reset condition (right), the rat can reset the PD delay back to zero by entering the FD trough. In the no-reset condition (left), the PD delay continues to increase regardless of FD choice. In the reset condition, rats should switch between the FD and PD options prior to the PD delay reaching equality with the FD trough (10 s) to maximize the number of rewards they earn in a session.

The timeline for the experiment is provided in Figure 1A, and all task details are included in the following sections. Rats (*n* = 21) were initially trained on the stop-change task for approximately 4-weeks prior to 10 days of testing on the stop-change task. Training consists of 1–2 days of habituation, followed by a progressive shaping task that lasts 1–3 weeks. Rats were then exposed to the full stop-change task for 5 days prior to the start of testing. Following 10 days of stop-change testing, the same rats were trained on the diminishing returns task for 4 weeks. Rats were then tested on the full diminishing returns task with reversals for 15 days. Following the completion of testing on both tasks (i.e., Experiment 1), rats were housed individually and interacted with daily for 11–12 months before undergoing an additional 12 days of testing on the stop-change task and 15 days of testing on the diminishing returns task (i.e., Experiment 2). During the 11–12 months between experiments, all 9 rats from the original 2–3-month-old cohort survived and were tested on Experiment 2. However, 4 of the rats from the original 9–11-month-old cohort died leaving us with 8 rats for the 21–23-month timepoint. In Experiment 3, rats, regardless of age from Experiment 1 and 2, were combined into a single cohort (*n* = 17) for examination of the impact of HDAC5 overexpression (HDAC5 +) in ACC and were randomly assigned to either control (GFP) or HDAC5 + conditions. We excluded one HDAC5 + rat from analysis following the immunoblotting results which failed to detect a greater than 120% increase in protein expression compared with controls. We excluded four additional rats due to complications with surgery, during recovery or during testing. Together, this left us with a total of 12 rats for Experiment 3 (GFP: *n* = 6; HDAC5 + : *n* = 6) for final analysis. Following 2 weeks of surgery and recovery, rats were again tested on the stop-change task for 15 days and diminishing returns task for 10 days. The changes in sample size and treatment groups are reflected in the timeline for Figure 1A. All behavioral testing sessions, regardless of behavioral task, lasted 1 h and rats were free to perform as many trials as they could within that time.

### Stop-change task

The task design is illustrated in Figure 1B. Each trial began with illumination of a house light that instructed the rat to nose poke into a central port. Nose poking initiated a 1,000 ms pre-cue delay period. It is important to note that poking into the central port does not obstruct the rats view of either cue light. At the end of this delay, a directional light to the rat’s left or right was flashed for 100 ms. If the rat exited the port at any time before the offset of the directional cue light, the trial was aborted, and house lights were extinguished. On 80% of trials (GO trials), presentation of the left or right light signaled the direction in which the rat should respond in order to obtain a 10% sucrose solution reward in the corresponding fluid well below. On the remaining 20% of trials (STOP trials), the light opposite to the location of the originally cued direction turned on either at the same time as port exit or after a randomly selected stop-signal delay (0–100 ms) and remained illuminated until the behavioral response was made. On STOP trials, rats were required to inhibit the movement signaled by the first light and respond in the direction of the second light. GO and STOP trials were randomly interleaved. On correct responding trials, rats were required to remain in the fluid well for a variable period between 800–1,000 ms (pre-fluid delay) before reward delivery (10% liquid sucrose solution). Error trials (incorrect direction) were immediately followed by the extinction of house lights and inter-trial-interval (ITI) onset of 4 s. The ratio of GO (80%) to STOP (20%) trials is intentional in order to ensure that rats develop a prepotent response to the first cue, thus making STOP trials surprising, and more likely to recruit inhibitory control processes (Verbruggen et al., 2019).

Trials were presented in a pseudorandom sequence such that left and right trials were presented in roughly equal numbers. The time necessary to stop and redirect a motor action [top-change reaction time (SCRT)] on STOP trials was computed using the difference between the average movement time on correct STOP and GO trials (Brockett and Roesch, 2021a; Brockett and Roesch, 2021b; Brockett et al., 2020; Bryden and Roesch, 2015; Bryden et al., 2011; Bryden et al., 2012; Bryden et al., 2016; Bryden et al., 2019; Tennyson et al., 2018). While we recognize there are multiple ways to estimate the timing necessary to inhibit a movement (Verbruggen and Logan, 2008), we choose to use SCRT because we have access to STOP trial movement time distributions and we vary the stop-signal delay systematically across sessions, making SSRT-mean and integration methods inappropriate for our dataset (Verbruggen and Logan, 2008; Verbruggen et al., 2019).

### Diminishing returns task

All testing for the diminishing returns task took place in three identical modular shuttle boxes (ENV-010MD, Med Associates, St. Albans, VT). Each shuttle box was contained within a sound and light attenuating cabinet. The end wall of each box was equipped with a stimulus light, retractable lever, and pellet trough connected to a pellet dispenser (Med Associates ENV-221M, ENV11CM, ENV-200R2M-6, and ENV-203M-45, respectively). The food troughs were positioned on the left half of the end-walls, when facing the wall from inside the chamber. The retractable levers were positioned on the lower right wall, such that when the levers extended, they were approximately 2 cm above the grid floor (Med Associates ENV010MB-GF). The stimulus light was positioned immediately above the levers. Lever assignments were randomized across rats, to minimize the impact of bias.

During performance of the diminishing returns task (Figure 1C), rats chose between two troughs that would deliver reward on different temporal schedules (Schuweiler et al., 2021). Each trial began with the insertion of both levers into the box. When one lever was pressed, both levers retracted, and the cue light located above the pressed lever was illuminated until 2 s after reward was delivered to the adjacent food trough. If a rat did not press a lever within 30 s of its extension, the trial was recorded as an omission, both levers were retracted, and a 30-s ITI was initiated. One trough delivered a pellet at a fixed delay (FD) of 10 s, while the other delivered a pellet immediately, but progressively increased (PD) its delay by 1 s every time it was selected (max 50 s). During “reset” sessions, selection of the FD trough reset the delays in the PD trough.

During no-reset sessions, rats should choose the PD trough until it reaches a delay of 10 s and then choose the FD trough for the remainder of the session to maximize reward over time (Figure 1C, left). During reset sessions, maximal reward over time is achieved when the average delay across the session in the PD trough is lower than 10 s. Optimal behavior is achieved by selecting the PD option 4 consecutive times and then enduring the 10 s delay in the FD trough to reset the PD delay back to zero (i.e., delayed gratification; Figure 1C, right). Roughly, over the course of the session, this corresponds to selecting the PD lever 80% of the time.

### HDAC5 overexpression

All surgical procedures followed guidelines for aseptic technique and were conducted in a dual arm rat stereotax (David Kopf Instruments, Tujunga, CA) fit with single microsyringe pump driver (WPI, Sarasota, FL). Rats were anesthetized with isoflurane (2–3%) and received bilateral infusions of either AAV2-CMV-GFP (*n* = 6) as a control or AAV2-CMV-mHDAC5-3SA (*n* = 7) to overexpress a nuclear-localized HDAC5 (HDAC5 +) into the ACC at the following coordinates relative to bregma: AP: + 0.2 mm, ML: ± 0.5 mm, DV: −2.2 mm. Coordinates were chosen based on previously published recording and lesion studies targeting the same region of ACC (Brockett et al., 2020; Bryden et al., 2019). All injections were performed with a 10 μl microsyringe with a blunt 33 ga needle (WPI, Sarasota, FL). Following injection, the needle sat undisturbed for 5–10 min before being slowly removed. Rats were given 10 days to recover before being tested on the stop-change task for an additional 15 days, and then the diminishing returns task for an additional 10 days. Rats were randomly assigned to GFP or HDAC5 + conditions such that performance did not differ between the two groups at the time of surgery. The use and effectiveness of the AAV2-CMV-mHDAC5-3SA in increasing nuclear localization of HDAC5 has been well-documented across several labs, including our own, and quantified previously (Li et al., 2018; Pribut et al., 2021; Taniguchi et al., 2017).

Following surgery, rats were administered Rimadyl (5 mg/kg) subcutaneously. Rats also received subcutaneous injections of Rimadyl (5 mg/kg), once daily for 2–3 days following surgery, and Cephalexin (15 mg kg^–1^, PO) was administered orally once per day for 7 days postoperatively. Following behavioral testing, rats were anesthetized with isoflurane (5%) and decapitated for immunoblotting analysis.

### Immunoblotting

Following decapitation, brains were rapidly extracted, flash frozen in 2-methyl butane (kept on dry ice) for 30 s, and stored at −80°C. Frozen brains were sectioned on a cryostat and 1-mm tissue punches were taken from ACC and placed in an Eppendorf tube on dry ice and stored at −80°C. For immunoblotting, tissue punches were quickly sonicated in lysis buffer and debris was removed by centrifugation as previously described (Boudreau et al., 2012). We processed all samples (20 μg per lane) for immunoblotting as previously described (Li X. et al., 2013) using the following antibodies: anti-HDAC5 (sc-133106, 1:500, Santa-Cruz, RRID:AB_2116793) and anti-histone 3 (H3, ab1792, 1:2000, Abcam, RRID:AB_302613). Blots were initially incubated in blocking buffer (5% non-fat dry milk in 1x Tris-buffered saline with 0.05% Tween-20) for 1 h at room temperature, followed by primary antibodies diluted in blocking buffer at 4°C overnight. The following day, we incubated blots with HRP-conjugated secondary antibodies (anti-mouse IgG, ThermoFisher Scientific, RRID:AB_2536527; anti-rabbit IgG, ThermoFisher Scientific, RRID:AB_1500696) diluted at 1:5,000 in blocking buffer for 1 h at room temperature. Blots were then imaged with an Azure c300 (Azure Biosystems). Signals were quantified using AzureSpot Image Analysis Software (Azure Biosystems). We used H3 as the loading control.

### Experimental design and statistical analysis

Behavior files were analyzed either using custom written code in MATLAB (Mathworks 2023b, Natick, MA), R,^1^ or Graphpad Prism 10.1 software (Graphpad Software, Boston, MA). For stop-change performance, percent correct scores were calculated by dividing the number of correct GO and STOP trials by the total number of trials. Reaction time values were generated by calculating the time from the first cue presentation to port exit. Movement time values were generated by calculating the time from center port exit to well entry. Planned *t*-tests were conducted, where appropriate, to verify the directionality of interactions. Unless otherwise specified, all behavioral data (i.e., percent correct or reaction time data) was analyzed using a two-way ANOVA, where each datum is a session average.

For the diminishing returns task, the first 5 days of testing served as the reminder days, where rats were reminded of the diminishing returns task after surgery or the passage of time. The second 5 days of testing served as a baseline pre-task variation reversal period. During these first 2 weeks, rats were tested on the last variation of the task they experienced during training. The final 5 days, during the third week of testing, served as the post task variation reversal period, where the task variation (reset or no-reset) was switched, and the PD trough and FD trough assignments were reversed.

To analyze the data, we computed separately for reset and no-reset sessions percentage of omitted trials, the total trials completed, the average choice on the PD trough and the average delay of the PD trough. For these behavioral measures, we performed a series of ANOVAs across sessions. Unless otherwise specified, all behavioral data was analyzed using a two-way ANOVA, where each datum is a session average.

## Results

### Differences in inhibitory control do not achieve significance when comparing 3–4-month and 10–12-month-old rats

Twenty-one rats (3–4 mo.: *n* = 9; 10–12 mo.: *n* = 12) were first trained and tested on the stop-change task, as shown in the experimental timeline in Figure 1A. In brief, rats began each trial by nose poking into the central port upon illumination of houselights. The center port, while recessed, is not deep enough to obscure visual access to either cue light that extend out past the wall of the behavioral panel. After 1,000 ms, one of two lights (left or right) was illuminated for 100 ms. On 80% of trials, rats responded in the direction of the light cue to obtain reward (GO trials). On 20% of trials, a second light cue was illuminated within 100 ms after the rat exited the central port. The ratio of GO (80%) to STOP (20%) trials is intentional in order to ensure that rats develop a prepotent response to the first cue (Verbruggen et al., 2019). During these “STOP-change” trials rats had to inhibit their initial movement in the direction of the first light and redirect their movement in the direction of the second light to obtain reward. For all trials, a reward was delivered 800–1,000 ms after entering the fluid well. In total, there were four possible trial-types: go-left, go-right, stop-left-go-right, and stop-right-go-left (Figure 1B).

**FIGURE 2:**
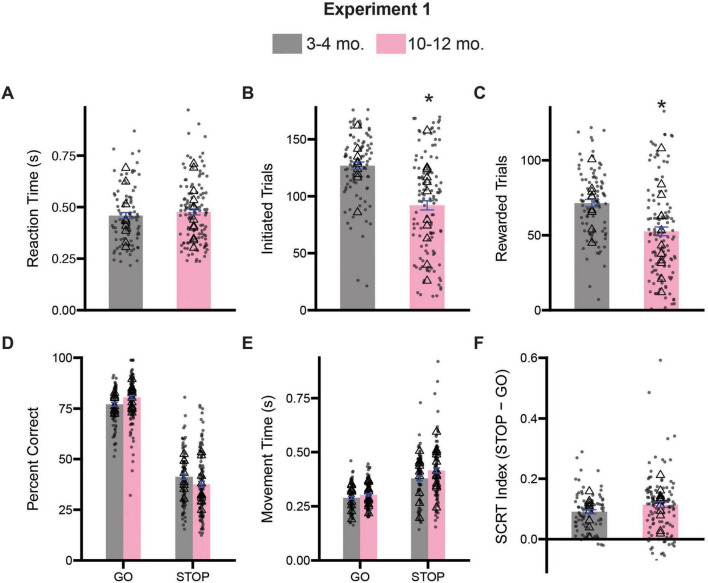
Comparison stop-change performance between 3–4- and 10–12-month-old rats. **(A)** Comparison of reaction times (the time from the first cue presentation to port exit). **(B)** Comparison of the number of initiated trials. **(C)** Comparison of the average number of rewarded trials. **(D)** Two-way ANOVA (trial type x treatment) assessing accuracy. **(E)** Two-way ANOVA (trial type x treatment) assessing movement time (i.e., the time from center port exit to well entry). **(F)** Stop-change reaction time (SCRT) index assessing overall inhibitor overall inhibitory control. Bars represent mean ± SEM. *Indicated *p* < 0.05. Dots represent session performance for each animal. Triangles represent animal averages.

We initially assessed whether motivation or task engagement measures differed between 3–4-month-old and 10–12-month-old rats. We observed no differences in reaction time, (i.e., the time from the first cue presentation to port exit) between groups suggesting that both groups responded to cue presentation with similar vigor [*t*(205) = 0.8619, *p* = 0.3898] (Figure 2A). We then examined the numbers of initiated trials and rewarded trials during the 60-min test sessions. We observed that 10–12-month-old rats initiated fewer trials [*t*(205) = 6.402, *p* < 0. 0001] and received fewer rewards [*t*(205) = 4.753, *p* < 0.0001] (Figures 2B,C). Collectively, these results suggest that on the stop-change task 3–4- and 10–12-month-old rats seem to respond to stimuli with similar levels of eagerness, however, we cannot rule out the possibility that older rats may become satiated earlier or are more apathetic to rewards generally as has also been described (Orsini et al., 2022). However, 10–12-month-old rats overall performed fewer trials and received less reward.

We next examined accuracy and movement time (i.e., the time from center port exit to well entry) performance as a function of trial-type. A 2-way ANOVA (trial-type x age) revealed an expected significant main effect of trial type for both percent correct [*F*(1,409) = 260.9, *p* < 0.0001] and movement time measures [*F*(1,407) = 117.5, *p* < 0.0001] suggesting that on average, rats were more accurate and faster on GO trials relative to STOP trials, consistent with the underlying assumption of the task (Figures 2D,E; Verbruggen et al., 2019). However, we only observed a significant main effect of age on our movement time measure [*F*(1,407) = 7.106, *p* = 0.0080], but not accuracy measure [*F*(1,409) = 3.288, *p* = 0.0705]. We observed no significant interaction between trial type and age for either accuracy [*F*(1,409) = 0.0973, *p* = 0.7533] or movement time measures [*F*(1,407) = 1.539, *p* = 0.2155] (Figures 2D,E). Collectively, we show that while 10–12-month-old rats are significantly slower at performing both GO and STOP trials, this does not significantly impact overall accuracy or inhibitory control. These findings are corroborated by the fact that an analysis of the inhibitory control index (SCRT) revealed no significant differences between cohorts at the 3–4- and 10–12-month timepoints [*t*(202) = 1.766, *p* = 0.0790] (Figure 2F).

Finally, to ensure that performance was not impacted by multiple days of testing, we re-analyzed percent correct and movement time data using a three-way ANOVA with session as a factor (session x trial type x age). The three-way ANOVA did not reveal a significant main effect of session for either percent correct [*F*(9,373) = 1.754, *p* = 0.0757] or movement time measures [*F*(9,371) = 0.8321, *p* = 0.5855]. Further, none of the possible interactions with session were significant (*p*’s > 0.05) for either measure suggesting that behavior was consistent across the testing period.

### Trial sequence effects demonstrate significant impairments in inhibitory control processes in 10–12-month-old rats

One advantage to the stop-change task is the ability to assess how experience on the previous trial impacts performance on future trials. Often the experience of conflict on STOP trials causes subjects to slow their behavior on the following trial (i.e., conflict adaptation) to increase the likelihood of performing the trial correctly. Conversely, when subjects experience multiple GO trials in a row, movement time measures tend to speed up. We examined the effect of trial order on accuracy and movement times in our 3–4-month and 10–12-month-old rats.

**FIGURE 3:**
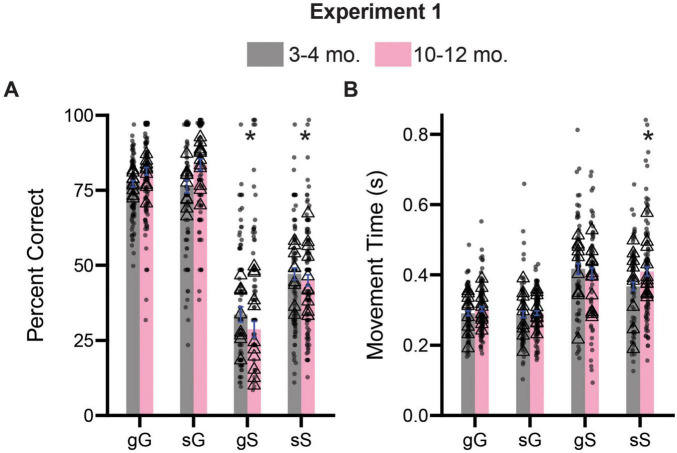
Comparison of trial experience on accuracy and movement times during Experiment 1. **(A)** Comparison of percent correct measures across gG, sG, gS, and sS trials. **(B)** Comparison of movement time measures across gG, sG, gS, and sS trials. Lower case “g” or “s” represent previous trial type (i.e., GO or STOP), upper case “G” or “S” represent current trial type (i.e., GO or STOP). Bars represent mean ± SEM. *Indicated *p* < 0.05. Dots represent session performance for each animal. Triangles represent animal averages.

To assess the effects of trial sequence on inhibitory control processes, we divided trials into their four possible combinations (gG: go on the previous trial, GO on the current trial; sG: stop on the previous trial, GO on the current trial; gS: go on the previous trial, STOP on the current trial, sS: stop on the previous trial, STOP on the current trial), and performed a three way ANOVA (previous trial type x current trial type x age) on percent correct and movement time measures. For percent correct, we observed a significant main effect of current trial type [*F*(1,796) = 86.9, *p* < 0.0001] and for age [*F*(1, 796) = 52.64, *p* < 0.0001], as well as a significant interaction between current trial type and age [*F*(1,796) = 10.41, *p* < 0.0001]. All other possible two and the three-way interactions were not significant (*p*’s > 0.05). Sidak-corrected *post hoc* comparisons revealed that when broken up by trial type, 10–12-month-old rats performed gS (*p* < 0.0001) and sS (*p* < 0.0001) trials significantly worse than 3–4-month-old rats (Figure 3A). We repeated this same analysis for movement time data in Figure 3B, and observed a significant main effect of current trial type [*F*(1,733) = 203.4, *p* < 0.0001], but no main effect of previous trial type [*F*(1,733) = 3.790, *p* = 0.0519] or age [*F*(1,733) = 2.226, *p* = 0.1361]. However, we did observe a significant three-way interaction (previous trial type x current trial type x age) [*F*(1,733) = 4.409, *p* = 0.0361]. Sidak-corrected *post hoc* comparisons revealed that 10–12-month-old rats were slower on sS trials relative to 3–4-month-old rats (*p* = 0.0395) (Figure 3B).

### Delayed gratification during reset sessions does not systematically differ between 3–4- and 10–12-month-old rats

The diminishing returns task illustrated in Figure 1C forces rats to choose between two offers, the FD offer which guarantees reward after a 10-s delay or the PD offer, which early on offers reward with minimal delay, but this delay increments with each selection. Self-control, or optimal decision-making, is assessed by comparing performance on no-reset and reset session types, where in the no-reset condition the PD lever continually increases with each press, however, in the reset condition selection of the FD lever, and enduring the 10 s wait, resets the PD lever back to 0. If rats recognize the difference between the two sessions-types, reset and no-reset, then they should select the PD trough at different rates. During no-reset sessions, rats should choose the PD trough until the delay reaches the equality point (i.e., 10 s) and then switch to the FD trough for the remainder of the session, thus during the no-rest sessions rats should choose PD lever far less often than the FD lever, and optimally the delay on the PD lever should not exceed 10 s (Figure 1C; left). Lever positions were counterbalanced across rats in order to minimize the impact of directional bias.

**FIGURE 4:**
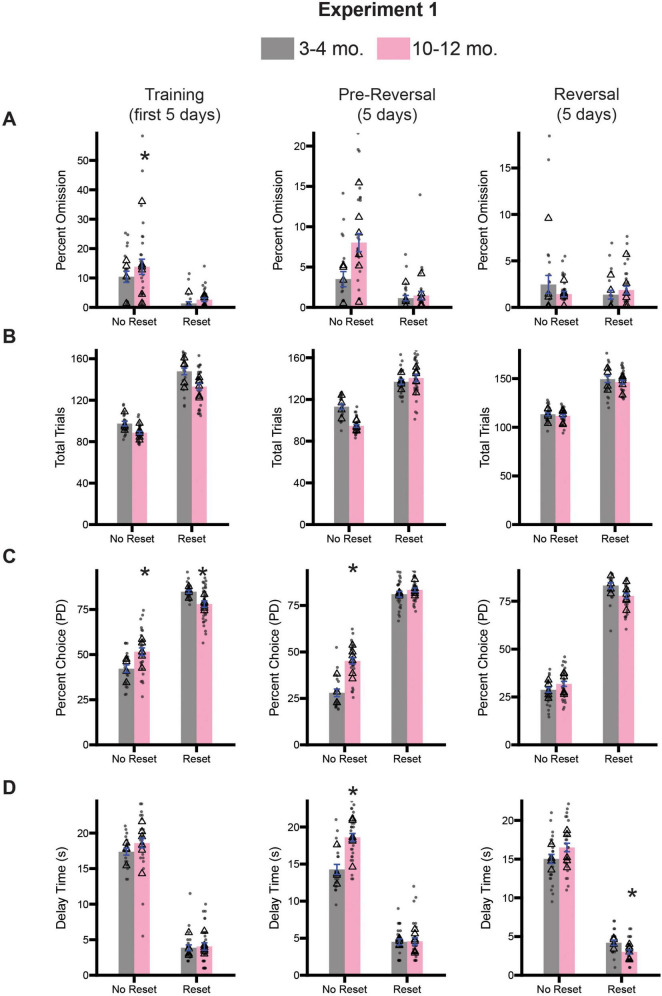
Comparison of diminishing return performance across training (days 1–5; left), pre-reversal (days 6–10; center), and reversal (days 11–15; right) for Experiment 1. **(A)** Comparison of percent omission for all three time points. **(B)** Comparison of the average number of trials performed for all three timepoints. **(C)** Comparison of the percentage of PD choices for all three timepoints. **(D)** Comparison of the average delay associated with pressing the PD for all three timepoints. Bars represent mean ± SEM. *Indicated *p* < 0.05. Dots represent session performance for each animal. Triangles represent animal averages.

Following 10 days of testing on the stop-change task, we trained rats on the diminishing returns task, which involved shaping lever press behavior and then teaching rats about each session-type. Half of the rats from both cohorts were assigned to begin testing on the no reset condition, while the other half began testing on the reset condition for 5 days (Training; Figure 4, left column). Rats continued with this session type for another 5 days (Pre-Reversal; Figure 4, center column), before being switched to the opposite session type for the final 5 days of testing (Reversal; Figure 4, right column). Inclusion of the reversal allows us to assess flexibility and ensure that rats can still learn new contingencies. As with the stop-change task, we assessed task engagement by calculating the percentage of omitted trials and total trials performed. For each measure and time point (i.e., Training, Pre-Reversal, Reversal), we performed a 2-way ANOVA (session type x age). During training we observed a significant main effect of session type [*F*(1,101) = 34.68, *p* < 0.0001] for percent omission, but no effect of age [*F*(1,101) = 1.843, *p* = 0.1777] or an interaction [*F*(1,101) = 0.3779, *p* = 0.5401] (Figure 4A). During the pre-reversal period we observed a significant main effect of session type [*F*(1,101) = 29.57, *p* < 0.0001] and age [*F*(1,101) = 8.748, *p* = 0.0039], as well as a significant interaction [*F*(1,101) = 6.552, *p* = 0.0120] (Figure 4A). Sidak-corrected *post-hoc* testing revealed a significant difference between age groups on the no reset condition, with 10–12-month-old rats omitting significantly more trials (*p* = 0.0005). However, during the reversal, a two-way ANOVA revealed no significant main effects for either session type [*F*(1,101) = 0.8187, *p* = 0.3677] or age [*F*(1,101) = 0.5015, *p* = 0.4805], nor a significant interaction [*F*(1,101) = 2.733, *p* = 0.1014] (Figure 4A).

We repeated this analysis for total trials during the same three time points. During training we observed a significant main effect of session type [*F*(1,101) = 318.5, *p* < 0.0001] and age [*F*(1,101) = 19.82, *p* < 0.0001], but no significant interaction [*F*(1,101) = 1.245, *p* = 0.2671] (Figure 4B). During the pre-reversal period we observed a significant main effect of session type [*F*(1,101) = 186.3, *p* < 0.0001] and age [*F*(1,101) = 8.483, *p* = 0.0044], as well as a significant interaction [*F*(1,101) = 18.82, *p* < 0.0001] (Figure 4B). Sidak-corrected *post-hoc* testing revealed a significant difference between age groups on the no reset condition, with 10–12-month-old rats omitting significantly more trials (*p* < 0.0001). However, during the reversal, a two-way ANOVA revealed only a significant main effect of session type [*F*(1,101) = 145.8, *p* < 0.0001], but no main effect of age [*F*(1,101) = 0.2347, *p* = 0.6291], or a significant interaction [*F*(1,101) = 1.66, *p* = 0.2038] (Figure 4B).

Collectively, the analysis of the percentage of omitted trials and total trials performed reveal small differences between 3–4-month-old and 10–12-month-old rats, suggesting that during the no-reset condition, 10–12-month-old rats may become less engaged by the task than 3–4-month-old rats. However, once there is a change in task conditions either at the beginning of the testing block or during the reversal, issues of task engagement seem to dissipate.

Next, to examine whether changes in session type impacted choice behavior, we performed similar analyses to the ones described before of the percent choice of the PD lever and the average delay time for choosing the PD lever. For percent choice, we observed a significant main effect of session type [*F*(1,101) = 346.9, *p* < 0.0001], but no main effect of age [*F*(1,101) = 0.4785, *p* = 0.4907], suggesting that rats regardless of age chose the PD lever more during the Reset condition (Figure 4C). However, we also observed a significant interaction between session type and age [*F*(1,101) = 19.23, *p* < 0.0001]. Sidak-corrected *post hoc* comparison revealed that during the No Reset condition 10–12-month-old rats selected the PD lever more than 3–4-month-olds (*p* = 0.0115). During the reset condition the effect was the opposite (*p* = 0.0162) suggesting subtle differences in optimal choice behavior during this time. During the pre-reversal period we also observed significant main effects of session type [*F*(1,101) = 822.6, *p* < 0.0001], age [*F*(1,101) = 37.59, *p* < 0.0001], and a significant interaction between them [*F*(1,101) = 21.66, *p* < 0.0001] (Figure 4C). However, Sidak-correct *post hoc* comparison only revealed a significant difference in percent choice between the age groups during the No Reset condition, with 10–12-month-old rats choosing the lever more often (*p* < 0.0001). Following reversal, we observed a significant main effect of session type [*F*(1,101) = 345.1, *p* < 0.0001], suggesting rats recognized the change in conditions, but no effect of age [*F*(1,101) = 3.189, *p* = 0.0771] or an interaction [*F*(1,101) = 0.0479, *p* = 0.8272] (Figure 4C).

We next examined average delay time on the PD lever across the three time periods. During training we observed a significant main effect of session type (F(1,101) = 606.7, *p* < 0.0001), but no main effect of age [*F*(1,101) = 1.508, *p* = 0.2223] or a significant interaction [*F*(1,101) = 0.7998, *p* = 3733] (Figure 4D). During the pre-reversal period we observed significant main effects of session type [*F*(1,101) = 506.0, *p* < 0.0001], age [*F*(1,101) = 17.29, *p* < 0.0001], and a significant interaction between them [*F*(1,101) = 16.25, *p* < 0.0001] (Figure 4D). Sidak-correct *post hoc* comparison only revealed a significant difference in average delay times between the age groups during the No Reset condition, with 10–12-month-old rats incurring a longer delay (*p* < 0.0001). Following reversal, we observed a significant main effect of session type [*F*(1,101) = 243.7, *p* < 0.0001], suggesting rats recognized the change in conditions, as well as a significant interaction [*F*(1,101) = 17.30, *p* < 0.0001] (Figure 4D). However, we observed no significant effect of age [*F*(1,101) = 1.690, *p* = 0.1965]. Sidak-correct *post hoc* comparison revealed that during the Reset condition 10–12-month-old rats experienced shorter average delays on PD relative to 3–4-month-old rats (*p* = 0.0003).

Overall, these results suggest that earlier in testing older rats were more willing to wait for delayed reward on no-reset sessions (Figure 4C Training and Pre-Reversal) as reported previously, and that during training, aged rats did not reset as often as younger rats (Beas et al., 2013; Hernandez et al., 2017; Roesch et al., 2012a; Roesch et al., 2012b; Setlow et al., 2009; Simon et al., 2010). However, through the course of the next 10 days, older rats reset at similar rates and experienced fewer delays to reward. Combined, maintaining responding of the PD lever that resulted in longer delays during no-reset, and the ability to endure the 10-s delay to reset the reward to experience short delays on the PD lever might reflect less sensitivity to delayed reward or potentially suboptimal decision-making, however neither of these were observed during reset sessions.

### Deficits in inhibitory control processes as measured by accuracy and movement time were observed later in life

Following the conclusion of testing on the diminishing returns testing, both cohorts of rats were maintained in individually housed cages for 11 months before the beginning of Experiment 2 (*see* Figure 1A). During this break, rats were handled daily and maintained on *ad libitum* food and water. Four of the twelve rats from the 10–12-month-old cohort in Experiment 1 passed away due to natural causes, as assessed by the University of Maryland’s veterinarian. In the following experiments, the 3–4-month-old cohort from Experiment 1 will be referred to as the 14–15-month-old cohort (*n* = 9), and the 10–12-month-old cohort from Experiment 1 will be referred to as the 21–23-month-old cohort. Rats from both cohorts were tested on both the stop-change task and the diminishing returns task with the same parameters that were used in Experiment 1.

**FIGURE 5:**
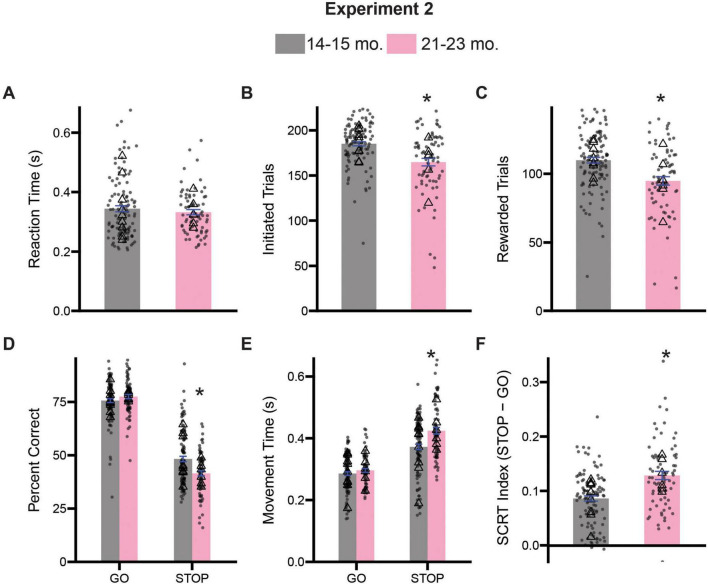
Comparison stop-change performance between 14–15- and 21–23-month-old rats. **(A)** Comparison of reaction times (the time from the first cue presentation to port exit). **(B)** Comparison of the number of initiated trials. **(C)** Comparison of the average number of rewarded trials. **(D)** Two-way ANOVA (trial type x treatment) assessing accuracy. **(E)** Two-way ANOVA (trial type x treatment) assessing movement time (i.e., the time from center port exit to well entry). **(F)** Stop-change reaction time (SCRT) index assessing overall inhibitor overall inhibitory control. Bars represent mean ± SEM. *Indicated *p* < 0.05. Dots represent session performance for each animal. Triangles represent animal averages.

Rats were tested on the stop-change task for 12 days. As before, we first assessed measures of task engagement and motivation by assessing the rat’s reaction times, number of initiated trials, number of rewarded trials and the number of error trials (Figures 5A–C). *T*-tests comparing average reaction times revealed no significant difference in responsiveness to cue presentation between 14–15- and 21–23-month-old rats [*t*(178) = 0.8177, *p* = 0.4146] (Figure 5A). Comparison of the number of initiated trials (Figure 5B) and the number of rewarded trials (Figure 5C) revealed that relative to 14–15-month old rats, 21–23-month old rats initiated fewer trials [*t*(178) = 4.593, *p* < 0.0001] and subsequently received fewer rewards [*t*(178) = 4.206, *p* < 0.0001], similar to the results in Experiment 1 (see Figures 2B,C).

Next, we performed two-way ANOVAs (trial type x age) on measures of accuracy and movement times to better assess aspects of inhibitory control. Analysis of the percentage of correct responses on both GO and STOP trials revealed significant main effects of trial type [*F*(1,356) = 776.3, *p* < 0.0001] and age [*F*(1,356) = 4.579, *p* = 0.0330], as well as a significant interaction between trial type and age [*F*(1,356) = 14.41, *p* = 0.0002]. Tukey-corrected *post hoc* comparisons revealed that 21–23-month-old rats were significantly worse at STOP (*p* = 0.0002), but not GO trials (*p* = 0.6458), relative to 14–15-month-old animals (Figure 5D). Analysis of the speed with which rats moved from the center port to the fluid well revealed significant main effects of trial type [*F*(1,356) = 171.1, *p* < 0.0001] and age [*F*(1,356) = 14.63, *p* = 0.0002], as well as a significant interaction [*F*(1,356) = 6.662, *p* = 0.0102]. Once again, Tukey-corrected *post hoc* comparisons revealed that 21–23-month-old rats were slower on STOP (*p* < 0.0001), but not GO (*p* = 0.8156), trials relative to 14–15-month-old rats (Figure 5E). A decrease in accuracy and movement time measures on STOP trials, specifically, suggests an inability to adapt or update an impending maladaptive response in the face of contradictory sensory information, and is a critical component of inhibitory control (Brockett and Roesch, 2021b; Verbruggen et al., 2019). We also computed a stop-change reaction time index as a composite measure of inhibitory control and found 21–23-month-old rats were significantly impaired compared to 14–15-month-old rats [*t*(178) = 5.015, *p* < 0.0001] (Figure 5F).

Finally, to ensure that performance was not impacted by multiple days of testing, we re-analyzed percent correct and movement time data using a three-way ANOVA with session as a factor (session x trial type x age). The three-way ANOVA did not reveal a significant main effect of session for our percent correct measure [*F*(11,312) = 0.4670, *p* = 0.9227], and all possible interactions with session were not significant (*p*’s > 0.05). However, we did observe a significant main effect of session on our movement time measures [*F*(11,312) = 2.092, *p* = 0.0206], but no interactions with session were significant (*p*’s > 0.05). This effect is not inherently surprising as across species, animals have shown a tendency to speed up responding overall with repeated trials/testing (Verbruggen et al., 2019). Further, the fact that none of the interactions with session were significant suggests that changes in movement time were equally distributed across the groups and trials.

### 21–23-month-old rats show impairments on sequence level measures of inhibitory control

**FIGURE 6:**
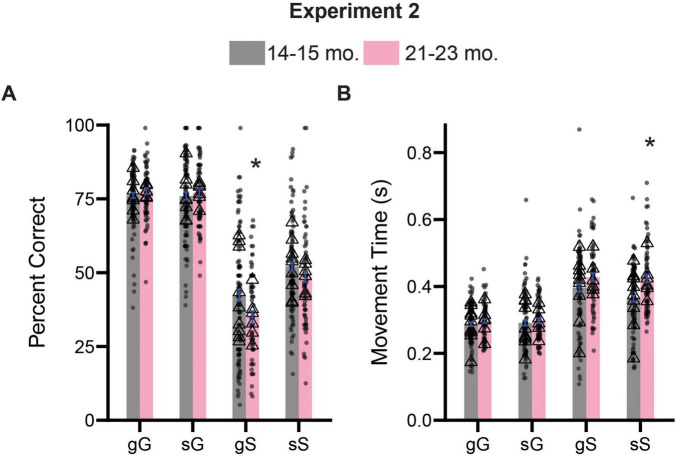
Comparison of trial experience on accuracy and movement times during Experiment 2. **(A)** Comparison of percent correct measures across gG, sG, gS, and sS trials. **(B)** Comparison of movement time measures across gG, sG, gS, and sS trials. Lower case “g” or “s” represent previous trial type (i.e., GO or STOP), upper case “G” or “S” represent current trial type (i.e., GO or STOP). Bars represent mean ± SEM. *Indicated *p* < 0.05. Dots represent session performance for each animal. Triangles represent animal averages.

As in Experiment 1, we were curious about the potential trial level differences in the performance of our rats on the stop-change task. We examined percent correct and movement time measures for the four possible sequence combinations described previously. A three-way ANOVA (previous trial type x current trial type x age) on accuracy measures revealed significant main effect of previous trial type [*F*(1,711) = 25.39, *p* < 0.0001] and current trial type [*F*(1,711) = 822.5, *p* < 0.0001], but not age [*F*(1,711) = 3.489, *p* = 0.0622], suggesting that accuracy measures in both groups were now influenced by the identity of both the previous and current trial type. We also observed a significant interaction between current trial type and age [*F*(1,711) = 9.084, *p* = 0.0027]. Sidak-corrected *post hoc* comparisons revealed that 21–23-month-old rats were significantly worse at gS trials (*p* = 0.0107), trials that require rats to suddenly STOP after previously experiencing a GO trial (Figure 6A). No other trial level differences were observed for our accuracy measure (*p*’s > 0.05).

Next, we performed a similar three-way ANOVA (previous trial type x current trial type x age) on our movement time measures. We observed a significant main effects for both current trial type [*F*(1,704) = 264.3, *p* < 0.0001] and age [*F*(1,704) = 16.53, *p* < 0.0001], as well as an significant interaction between both factors [*F*(1,704) = 8.047, *p* = 0.0047]. No other main effect or possible interactions were significant (*p*’s > 0.05). Sidak-corrected *post hoc* comparisons revealed that 21–23-month-old rats were significantly slower on sS trials (*p* < 0.0001) (Figure 6B).

Collectively, these findings largely support the results presented in the previous section and suggest that specific impairments in inhibitory control associated with the 21–23-month-old period result from deficits in stopping abilities.

### 21–23-month-old rats reset during diminishing returns similar to 14–15-month-old rats

**FIGURE 7:**
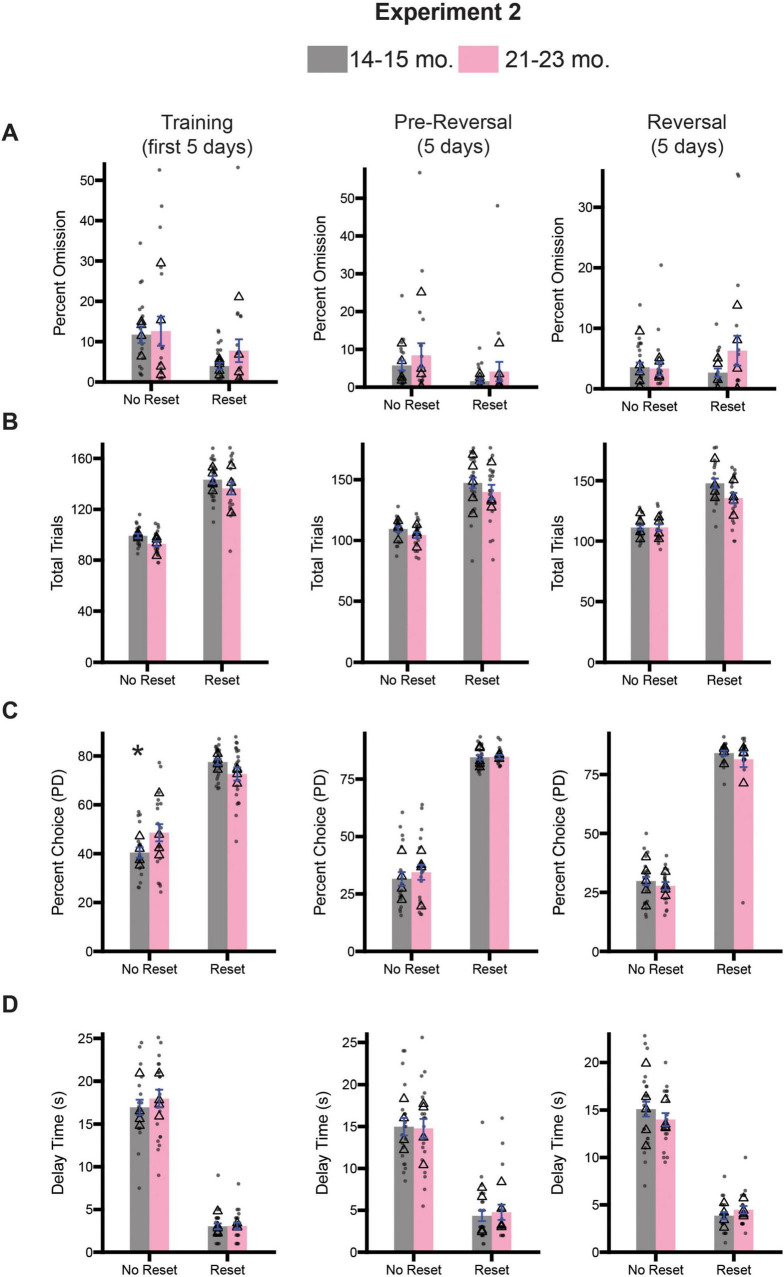
Comparison of diminishing return performance across training (days 1–5; left), pre-reversal (days 6–10; center), and reversal (days 11-15; right) for Experiment 2. **(A)** Comparison of percent omission for all three time points. **(B)** Comparison of the average number of trials performed for all three timepoints. **(C)** Comparison of the percentage of PD choices for all three timepoints. **(D)** Comparison of the average delay associated with pressing the PD for all three timepoints. Bars represent mean ± SEM. *Indicated *p* < 0.05. Dots represent session performance for each animal. Triangles represent animal averages.

Given the observed deficits in inhibitory control associated with the 21–23-month-old timepoint, we were curious to know whether age-related changes in optimal decision-making were also evident. Following the 10 days of testing on the stop-change task, both cohorts of rats underwent testing on the diminishing returns task. As before, half of the rats from both cohorts were assigned to begin testing on the no reset condition, while the other half began testing on the reset condition for 5 days (Training; Figure 7, left column). Rats continued with this session type for another 5 days (Pre-Reversal; Figure 7, center column), before being switched to the opposite session type for the final 5 days of testing (Reversal; Figure 7, right column). We assessed task engagement by calculating the percentage of omitted trials and total trials performed. For each measure and time point (i.e., Training, Pre-Reversal, Reversal), we performed a 2-way ANOVA (session type x age). During training we observed a significant main effect of session type [*F*(1,81) = 8.731, *p* = 0.0112] for percent omission, but no effect of age [*F*(1,81) = 0.9253, *p* = 0.3389] or an interaction [*F*(1,81) = 0.3649, *p* = 0.5475] (Figure 7A). During the pre-reversal period we observed a significant main effect of session type [*F*(1,80) = 4.261, *p* = 0.0422] as well, but no significant effect of age [*F*(1,80) = 1.689, *p* = 0.2041] or a significant interaction between both factors [*F*(1,80) = 0.0014, *p* = 0.9701] (Figure 7A). During the reversal a two-way ANOVA revealed no significant main effects for either session type [*F*(1,75) = 0.4797, *p* = 0.4907] or age [*F*(1,75) = 1.347, *p* = 0.2495], nor a significant interaction [*F*(1,75) = 1.683, *p* = 0.1985] (Figure 7A).

We repeated this analysis for total trials during the same three time points. During training we observed a significant main effect of session type [*F*(1,81) = 198.9, *p* < 0.0001] and age [*F*(1,81) = 4.552, *p* < 0.0359], but no significant interaction [*F*(1,81) = 0.0020, *p* = 0.9642] (Figure 7B), suggesting that 21–23-month-old rats did fewer trials overall regardless of session type. During the pre-reversal period we observed a significant main effect of session type [*F*(1,80) = 81.01, *p* < 0.0001], but not age [*F*(1,80) = 2.338, *p* = 0.1302]. We also did not observe a significant interaction between both factors [*F*(1,80) = 0.0640, *p* = 0.7727] (Figure 7B). During the reversal, a two-way ANOVA revealed only a significant main effect of session type [*F*(1,80) = 3.695, *p* < 0.0001], but no main effect of age [*F*(1,80) = 3.637, *p* = 0.0601], or a significant interaction [*F*(1,80) = 3.695, *p* = 0.0581] (Figure 7B). Collectively, both analyses suggest relatively small differences in measures of task engagement between 14–15-month-old and 21–23-month-old rats.

Next, we examined whether changes in session type impacted choice behavior. For percent choice of PD we performed a two-way ANOVA (session type x age), and observed a significant main effect of session type [*F*(1,81) = 157.6, *p* < 0.0001], but no main effect of age [*F*(1,81) = 0.4475, *p* = 0.5054], suggesting that rats regardless of age chose the PD lever more during the Reset condition (Figure 7C). We also observed a significant interaction between session type and age [*F*(1,81) = 7.147, *p* = 0.0091]. Sidak-corrected *post hoc* comparison revealed that during the No Reset condition 21–23-month-old rats selected the PD lever more frequently than 14–15-month-olds (*p* = 0.0471). During the pre-reversal period we observed significant main effects of session type [*F*(1,80) = 541.5, *p* < 0.0001], but no effect of age [*F*(1,80) = 0.4798, *p* = 0.4905], or a significant interaction between them [*F*(1,80) = 0.3592, *p* = 0.5506] (Figure 7C). Similarly, following reversal, we observed a significant main effect of session type [*F*(1,80) = 588.3, *p* < 0.0001], suggesting rats recognized the change in conditions, but no effect of age [*F*(1,75) = 1.085, *p* = 0.3010] or an interaction [*F*(1,75) = 0.0196, *p* = 0.8891] (Figure 7C).

Next, we examined average delay time across the three time periods. During training we observed a significant main effect of session type [*F*(1,81) = 419.3, *p* < 0.0001], but no main effect of age [*F*(1,81) = 0.5864, *p* = 0.4453] or a significant interaction [*F*(1,81) = 0.4747, *p* = 4928] (Figure 7D). During the pre-reversal period we observed a significant main effect of session type [*F*(1,80) = 124.2, *p* < 0.0001], but not age [*F*(1,80) = 0.0146, *p* = 0.9044]. We also did not observe a significant interaction between both factors [*F*(1,80) = 0.1181, *p* = 0.7375] (Figure 7D). Following reversal, we observed a significant main effect of session type [(1,75) = 256.2, *p* < 0.0001], suggesting rats recognized the change in conditions (Figure 7D). However, we observed no significant effect of age [*F*(1,75) = 0.1339, *p* = 0.7154] or a significant interaction between both factors [*F*(1,75) = 1.766, *p* = 0.1679].

Much like the results presented in Experiment 1, we did find some differences in specific measures between cohorts across the various stages of the task. Additionally, what could have been seen as a potential sign on suboptimal decision making in Experiment 1, never fully manifested in older rats in Experiment 2. Ultimately, these differences do not appear to be systematic nor suggestive of a global deficit in decision-making abilities. Instead, the vast majority of the findings suggest that 21–23-month-old rats retain, or possibly slightly improve, their reasoning/self-control abilities during performance of diminishing returns.

### HDAC5 overexpression further impairs inhibitory control processes in aged animals

The accumulation of epigenetic modifications over the course of the lifetime has been suggested to contribute to age-related decline in cognition and generally functioning (Dhar et al., 2022; Fischer et al., 2010; Kane and Sinclair, 2019; McIntyre et al., 2019; Pasyukova and Vaiserman, 2017; Wang et al., 2022). Histone deacetylases (HDACs) are a class of proteins responsible for cleaving acetyl groups from histone and non-histone proteins facilitating the tighter coiling of chromatin. Four major classes of HDACs exist, and the targeting of these proteins with HDAC inhibitors has been attributed to lifespan extension (Dhar et al., 2022; Kane and Sinclair, 2019; McIntyre et al., 2019; Pasyukova and Vaiserman, 2017; Wang et al., 2022). While growing evidence implicates HDACs in aging processes, few studies have investigated the impact of specific HDACs on inhibitory control and optimal decision-making. Here, we used AAV2-CMV-mHDAC5-3SA to overexpress a nuclear-localized HDAC5, a class IIA HDAC that generally suppresses gene expression, in the ACC of aged rats. Previous work has suggested that overexpression of HDAC5 in rat dorsal lateral striatum promotes inflexible decision-making (Pribut et al., 2021), so we were curious whether HDAC5 overexpression in ACC would reduce inhibitory control and optimal decision-making abilities.

**FIGURE 8:**
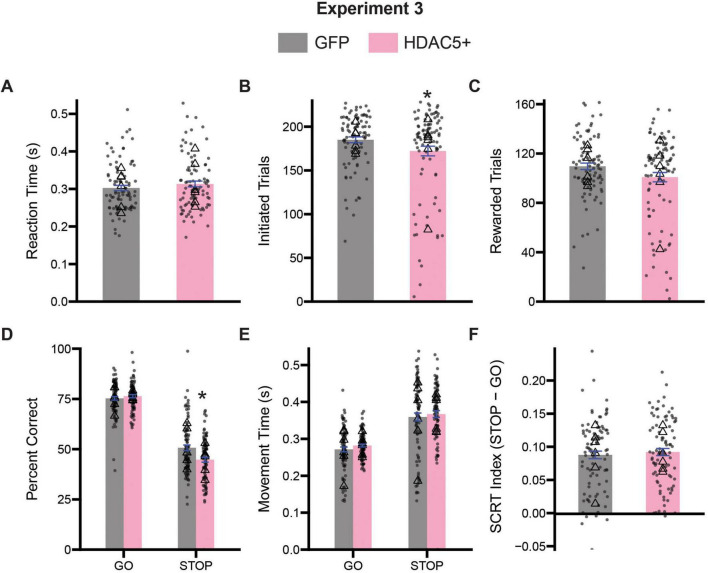
Comparison stop-change performance between GFP and HDAC5 overexpress (HDAC5 +) rats. **(A)** Comparison of reaction times (the time from the first cue presentation to port exit). **(B)** Comparison of the number of initiated trials. **(C)** Comparison of the average number of rewarded trials. **(D)** Two-way ANOVA (trial type x treatment) assessing accuracy. **(E)** Two-way ANOVA (trial type x treatment) assessing movement time (i.e., the time from center port exit to well entry). **(F)** Stop-change reaction time (SCRT) index assessing overall inhibitor overall inhibitory control. Bars represent mean ± SEM. *Indicated *p* < 0.05. Dots represent session performance for each animal. Triangles represent animal averages.

Successful overexpression of HDAC5 was determined by western blot. Rats transfected with nuclear-localized HDAC5 showed a 124.6% fold increase in HDAC5 levels on average relative to GFP controls [*t*(10) = 2.472, *p* = 0.0330] (Figure 1A). We set a threshold for fold change of HDAC5 mRNA expression to be > 120%, and eliminated one rat from all HDAC5 analyses that did not meet this criterion. Rats were tested on the stop-change task for 15 days. As before, we first assessed measures of task engagement and motivation by assessing the rat’s reaction times, number of initiated trials, number of rewarded trials and the number of error trials (Figures 8A–C). *T*-tests comparing average reaction times revealed no significant difference in responsiveness to cue presentation between GFP and HDAC5 + rats [*t* (177) = 1.037, *p* = 0.3012] (Figure 8A). Comparison of the number of initiated trials [*t*(177) = 2.001, *p* = 0.0469] (Figure 8B) did reveal a slight decrease in the number of initiated trials by HDAC5 + rats, and a trend, but ultimately not significant difference, in the number of rewarded trials [*t*(177) = 1.919, *p* = 0.0566] (Figure 8C) between GFP and HDAC5 + rats.

Next, we performed two-way ANOVAs (trial type x treatment) on measures of accuracy and movement times to better assess aspects of inhibitory control. Analysis of the percentage of correct responses on both GO and STOP trials revealed a significant main effect of trial type [*F*(1,354) = 639.8, *p* < 0.0001] and treatment [*F*(1,354) = 4.728, *p* = 0.0303], as a significant interaction between trial type and treatment [*F*(1,354) = 9.915, *p* = 0.0018]. Sidak-corrected *post hoc* comparisons revealed that HDAC5 overexpressing rats were significantly worse at STOP (*p* = 0.0004), but not GO trials (*p* = 0.7411), relative to GFP treated animals (Figure 8D). Analysis of the speed with which rats moved from the center port to the fluid well revealed significant main effect of trial type [*F*(1,354) = 116.9, *p* < 0.0001] suggesting that rats from both groups were slower on STOPs than GOs. However, we did not observe a significant main effect of treatment [*F*(1,354) = 1.338, *p* = 0.2482], nor a significant interaction [*F*(1,354) = 0.0246, *p* = 0.8755] (Figure 8E). We computed a stop-change reaction time index as a composite measure of inhibitory control and found HDAC5 overexpression did not significantly impair this measure [*t*(177) = 0.5579, *p* = 0.5776] (Figure 8F).

Finally, to ensure that performance was not impacted by multiple days of testing, we re-analyzed percent correct and movement time data using a three-way ANOVA with session as a factor (session x trial type x treatment). The three-way ANOVA did not reveal a significant main effect of session for our percent correct measure [*F*(14,297) = 0.4763, *p* = 0.9447], and all possible interactions with session were not significant (*p*’s > 0.05). We did observe a trend toward significance with our movement time measures as a function of session [*F*(14,298) = 1.692, *p* = 0.0564], but no interactions with session were significant (*p*’s > 0.05), suggesting that across treatment groups animals tended to speed up responding slightly with repeated testing. However, with both the two-way and three-way analyses no significant differences other than trial type were observed with regards to movement time.

### HDAC5 overexpression alters sequence level measures of inhibitory control

**FIGURE 9:**
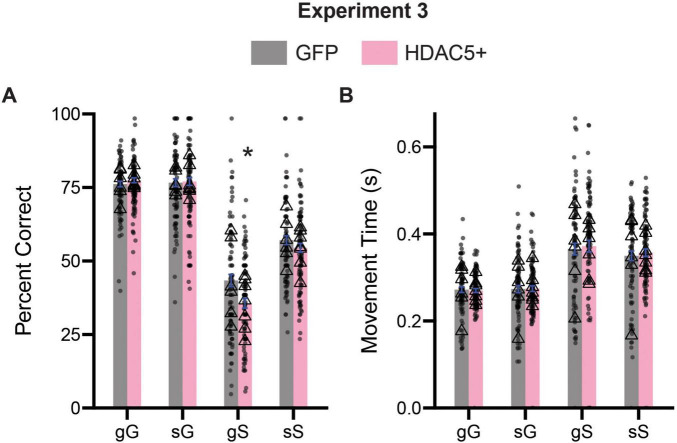
Comparison of trial experience on accuracy and movement times during Experiment 3. **(A)** Comparison of percent correct measures across gG, sG, gS, and sS trials. **(B)** Comparison of movement time measures across gG, sG, gS, and sS trials. Lower case “g” or “s” represent previous trial type (i.e., GO or STOP), upper case “G” or “S” represent current trial type (i.e., GO or STOP). Bars represent mean ± SEM. *Indicated *p* < 0.05. Dots represent session performance for each animal. Triangles represent animal averages.

To assess the effects of trial sequence on inhibitory control processes, we divided trials into their four possible combinations (gG: go on the previous trial, GO on the current trial; sG: stop on the previous trial, GO on the current trial; gS: go on the previous trial, STOP on the current trial, sS: stop on the previous trial, STOP on the current trial), and performed a three-way ANOVA (previous trial type x current trial type x age) on percent correct and movement time data. For percent correct, we observed a significant main effect of previous trial type [*F*(1,701) = 64.46, *p* < 0.0001], current trial type [*F*(1,701) = 745.5, *p* < 0.0001] and treatment [*F*(1,701) = 7.176, *p* = 0.0076]. We also observed a two-way interaction between previous trial type and current trial type [*F*(1, 701) = 64.78, *p* < 0.0001], as well as a significant interaction between current trial type and treatment [*F*(1,701) = 6.814, *p* = 0.0092]. All other possible two and the three-way interactions were not significant (*p*’s > 0.05). Sidak-corrected *post hoc* comparisons revealed that when broken up by trial type, HDAC5 + rats performed gS (*p* < 0.0007) significantly worse than GFP controls (Figure 9A). We repeated this same analysis for movement time data in Figure 9B. Here, as in Figure 8E, we only observed a significant main effect of current trial type [*F*(1,707) = 140.4, *p* < 0.0001]. All other possible main effects and interactions were not significant (*p*’s > 0.05) (Figure 9B).

### HDAC5 overexpression minimally impacts diminishing returns task performance

**FIGURE 10:**
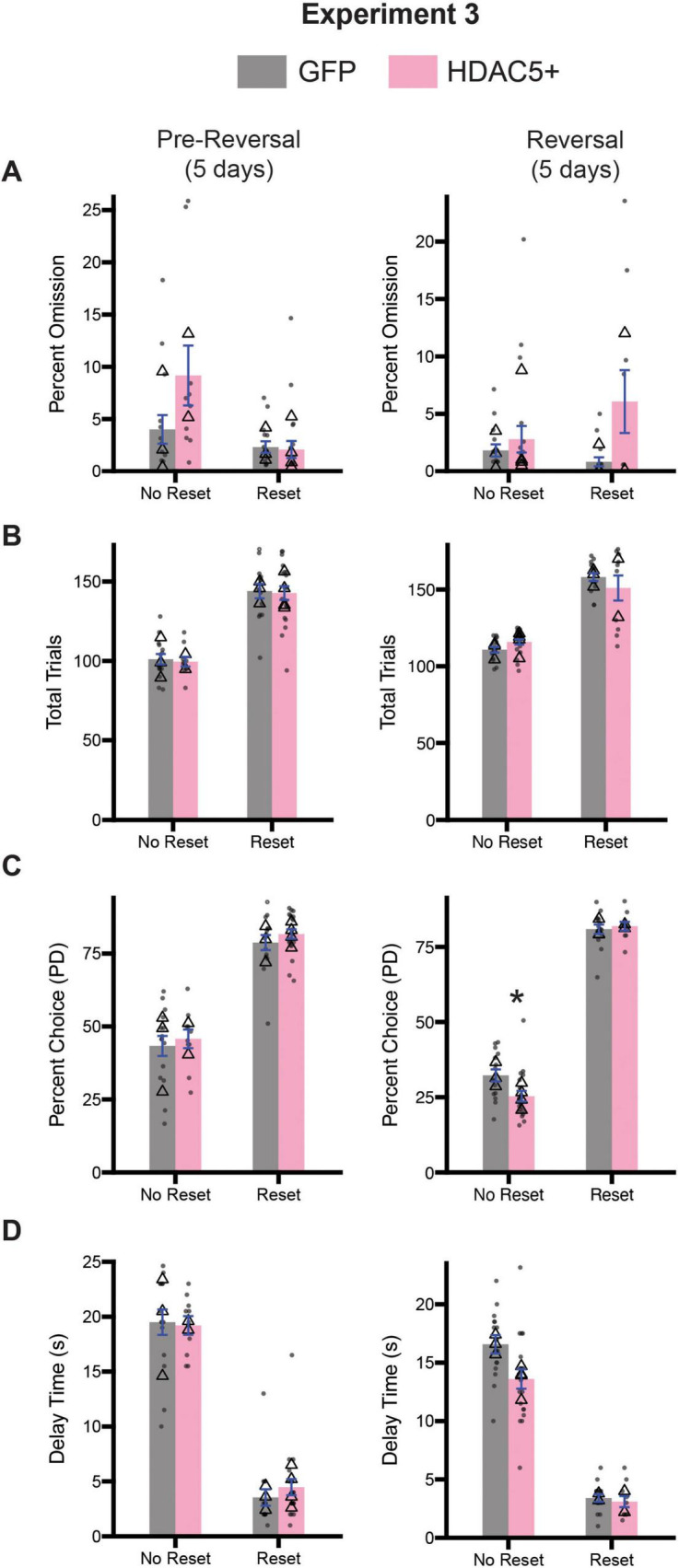
Comparison of diminishing return performance across pre-reversal (days 1–5; center, and reversal (days 6–10; right) for Experiment 3. **(A)** Comparison of percent omission for all three time points. **(B)** Comparison of the average number of trials performed for all three timepoints. **(C)** Comparison of the percentage of PD choices for all three timepoints. **(D)** Comparison of the average delay associated with pressing the PD for all three timepoints. Bars represent mean ± SEM. *Indicated *p* < 0.05. Dots represent session performance for each animal. Triangles represent animal averages.

Given the observed deficits in inhibitory control associated with HDAC5 overexpression, we were curious to know whether these changes would alter optimal decision-making behavior. Following the 15 days of testing on the stop-change task, both cohorts of rats underwent testing on the diminishing returns task. Half of the rats from both cohorts were assigned to begin testing on the no reset condition, while the other half began testing on the reset condition for 5 days (Pre-Reversal; Figure 10, left column), before being switched to the opposite session type for the final 5 days of testing (Reversal; Figure 10, right column). We assessed task engagement by calculating the percentage of omitted trials and total trials performed. For each measure and time point (i.e., Pre-Reversal and Reversal), we performed a 2-way ANOVA (session type x treatment). During the pre-reversal period we observed a significant main effect of session type [*F*(1,56) = 10.49, *p* = 0.0020], but no significant effect of treatment [*F*(1,56) = 3.303, *p* = 0.0745] or a significant interaction between both factors [*F*(1,56) = 3.958, *p* = 0.0515] (Figure 10A), suggesting that rats from both groups recognized the difference between no reset and reset conditions equally. During the reversal a two-way ANOVA revealed no significant main effect for session type [*F*(1,56) = 0.8125, *p* = 0.3713] nor a significant interaction [*F*(1,56) = 2.832, *p* = 0.0980], but we did observe a significant main effect of treatment [*F*(1,56) = 5.993, *p* = 0.0175] (Figure 10A). This suggests that HDAC5 + rats omitted more trials during the reversal period.

We repeated this analysis for total trials during the same time points. During the pre-reversal period we observed a significant main effect of session type [*F*(1,56) = 100.7, *p* < 0.0001], but not for treatment [*F*(1,56) = 0.1076, *p* = 0.7441] or the interaction between both factors [*F*(1,56) = 0.0014, *p* = 0.9707] (Figure 10B). During the reversal, a two-way ANOVA revealed a significant main effect of session type [*F*(1,56) = 144.5, *p* < 0.0001], but no main effect of treatment [*F*(1,56) = 0.0904, *p* = 0.7647] or a significant interaction between session type and treatment [*F*(1,56) = 3.152, *p* = 0.0813] (Figure 10B).

Next, we examined whether changes in session type impacted choice behavior. For percent choice of PD we performed a two-way ANOVA (session type x treatment), and observed a significant main effect of session type [*F*(1,56) = 173.6, *p* < 0.0001], but no main effect of treatment [*F*(1,56) = 0.9725, *p* = 0.3283] or a significant interaction between session type and age [*F*(1,56) = 0.0079, *p* = 0.9295] (Figure 10C). During the reversal period we observed significant main effects of session type [*F*(1,56) = 793.3, *p* < 0.0001], but no effect of treatment [*F*(1,56) = 2.520, *p* = 0.1180]. However, we did observe a significant interaction between session type and treatment [*F*(1,56) = 4.445, *p* = 0.0395] (Figure 10C). Tukey-corrected *post hoc* comparison revealed HDAC5 overexpression reduced PD presses during the no reset condition (p = 0.0280), but not during the reset condition (*p* = 0.9864) (Figure 10C).

Finally, we examined the average delay time across both time periods. During the pre-reversal, we observed a significant main effect of session type [*F*(1,56) = 278.9, *p* < 0.0001], but no main effect of treatment [*F*(1,56) = 0.1219, *p* = 0.7283] or a significant interaction [*F*(1,56) = 0.4565, *p* = 5021] (Figure 10D). During the reversal period we observed a significant main effect of session type [*F*(1,56) = 253.2, *p* < 0.0001] and treatment [*F*(1,56) = 4.824, *p* = 0.0322]. However, we did observe a significant interaction between both factors [*F*(1,56) = 3.215, *p* = 0.0784] (Figure 10D).

In summary, while we did find some differences on specific measures between cohorts across the various stages of the diminishing returns task, these differences were not systematic or suggestive of a global deficit in decision-making abilities. If anything, HDAC5 overexpression in the ACC made rats less sensitive to reward delays during no-reset sessions.

## Discussion

Across two experiments, separated by approximately 11 months, we evaluated the inhibitory control and self-control abilities of two cohorts of rats at various stages of life ranging from early adulthood to middle adulthood to advanced age. We show that relative to 3–4-month-old rats, 10–12-month-old rats began showing deficits on inhibitory motor control measures, but not self-control as measured by no-reset diminishing returns. In our second experiment, we showed that 21–23 months of age was associated with a significant decrease in inhibitory control abilities, at least in comparison to 14–15-month-old rats. However, despite the apparent loss of inhibitory control processes, 21–23-month rats showed very little loss in their ability to optimally reason about two distinct offers, suggesting that mechanisms of inhibitory control and decision-making abilities are distinct. Finally, in our third experiment we investigated the impacts of HDAC5 overexpression (HDAC5 +) in ACC on both measures of inhibitory control and self-control. We found that HDAC5 + worsened inhibitory control performance in aged rats, however, minimally impacted measures of self-control. Collectively, these findings characterize age-related changes in inhibitory control and self-control measures across the lifespan, while suggesting that inhibitory control processes, specifically in the ACC, are susceptible to the impact of aberrant accumulation of HDAC5.

Remarkably, once trained and regardless of age, rats readily performed both tasks and remembered how to perform each task at high levels even across the 11-month break in which they received no formal training or refresher sessions on either task. In many ways this approach also mimics normal human aging. While humans rarely perform multiple bouts of prescribed cognitive tasks as they age, the cognitive processes tested here are essential to healthy daily life. Age-related cognitive deficits typically emerge slowly such that abilities individuals were once readily capable of diminish with time. In that sense, by retesting rats repeatedly on the same cognitive tasks, our design imitates more daily usage of these cognitive processes. Additionally, our work helps provide a data-informed window of time in which interventions aimed at preserving cognitive abilities may be applied. Aging research in rodents typically focuses on the 21–23-month range, but this ignores the fact that age-related decline is often gradual (Bizon and Gallagher, 2003; Bizon et al., 2009). Based on our results, we would predict that inhibitory control processes begin to show some signs of decline at 10–12 months, which may be the ideal time to begin testing potential pharmacological or behavioral interventions, such as exercise, as a means to stave off age-related declines (Brockett et al., 2015).

Previous research using a multi-cohort study of young (6 months old), middle aged (12 months old), and aged (22 months old) rats demonstrated a progressive age-related decline in spatial reference and working memory measures in Fischer 344 rats (Bizon et al., 2009). Most importantly, the researcher found that middle-aged rats (12 months old) showed impairments in these measures relative to young (6 months old) rats, which is consistent with our current study. The authors suggest that both hippocampal and prefrontal regions may be implicated in this decline, and that healthy aging may alter the manner in which prefrontal regions are recruited to support behavior (Bizon et al., 2009). Our previous research using the same stop-change task has shown differential contributions of medial prefrontal cortex (mPFC) and anterior cingulate cortex (ACC) to inhibitory control (Brockett et al., 2020; Brockett et al., 2022; Bryden et al., 2019). Lesions to mPFC have been shown to slow behavior overall, impairing performance on GO trials, but improving performance on STOP trials (Brockett et al., 2022). Conversely, lesions to ACC have been shown to selectively impair STOP signal performance in a manner consistent with what was seen in 21–23 month old rats (Brockett et al., 2020). This raises the possibility that either the engagement of ACC or the structural and functional integrity of neurons in ACC may be compromised in healthy aging (Brockett and Roesch, 2021b; Brockett et al., 2020; Bryden et al., 2019).

Further, our results from Experiment 3 suggest that HDAC5 accumulation diminishes inhibitory control processes in aged rats. Importantly these effects were found in a sample of rats that were all aged, where the results from Experiment 2 suggest inhibitory control processes are already impaired. Identifying brain areas susceptible to the negative effects of epigenetic modifications may open doors to further insights into the mechanisms underlying age-related cognitive change.

Other work using Fischer 344 rats has shown that non-olfactory discrimination learning is maintained at least until 24 months of age, while olfactory discrimination learning is not (LaSarge et al., 2007). Much like our findings on optimal decision-making, these results collectively highlight that cognitive decline associated with healthy aging is not unitary and underscore the importance of examining multiple domains of cognition. The lack of systematic change in optimal decision-making abilities also mirrors findings in the human literature. Many studies have reported deficits in decision-making with age under novel or uncertain circumstances (Chowdhury et al., 2013; Mata et al., 2011; Samanez-Larkin and Knutson, 2015; Samanez-Larkin et al., 2007; Samanez-Larkin et al., 2012), while others suggest that when decision-making relies on more life experience, aged individuals perform quite well (Li Y. et al., 2013). Successful economic decision-making, like what is assessed in these human studies, likely draws on multiple cognitive domains. One way to conceptualize our findings in light of this work, is to suggest that older individuals with difficulties making decisions in light of novel information, may be reflective of age-related impairments in inhibitory control circuitries, however, when asked to decide between waiting a short amount of time versus a longer amount of time for food, something our rats have likely encountered several times in their lifetime, more experience overcomes any deficits in inhibitory control. This is at least somewhat supported by recording work in adolescent rodents that show adaptive firing patterns in frontal brain area to valanced reward information take time to develop into adult patterns (Loh and Rosenkranz, 2023).

Interestingly, in humans Lamichhane et al. (2020) showed that older adults earned fewer overall rewards than younger adults in a variant of the diminishing returns task. This deficit persisted even when the optimal strategy was explained, and was reported to be due to older subject’s self-reported difficulty in waiting for delayed rewards (Lamichhane et al., 2020). In our rats, we did not observe these difficulties, at least in a systematic manner, and this may be due to differences in the task structure or the fact that rats, regardless of age, were well-trained on our diminishing returns task.

Importantly, while all rats used in this study spent over half of their lives singly housed in our vivarium under identical living conditions, it is difficult to control the impact early life experience might have on their cognitive abilities across the lifespan. On the one hand, both cohorts were purchased from the same vendor, and were likely subject to similar housing, handling, and food. However, the original 10–12-month cohort were experienced breeders, and while this may enhance the ethological validity of our results, it could also present as a potential confound. Sexual experience has been associated with enhanced neuroplasticity, despite initial elevations in stress response (Leuner et al., 2010). It is possible that sexual experience may have blunted the negative impact of age on cognition from being detected earlier, however, given that deficits in inhibitory control were not as prominent at the 10–12 month and 14–15-month-old timepoints this concern is small. Additionally, rats were handled near daily for duration of the experiment. This experimenter interaction may have provided some enrichment that might have buffered against the negative effects of age (Balietti and Conti, 2022; Speisman et al., 2013), although here to, it is hard to assess the impact of social isolation which has been shown to negative impact brain health (Stranahan et al., 2006). What the human equivalent of this type of interaction is, is difficult to say, however, we think the ethologically validity of our approach is still useful as most humans maintain some level of interaction with others throughout their life. Nevertheless, this highlights the challenges in performing experiments like this across the lifespan.

In summary, we show that by studying two differently aged cohorts of rats at multiple time points on two tasks with clear translational relevance that inhibitory control and optimal decision-making are differentially impacted by age. Further, by capitalizing on the tractability of rodents, we demonstrated the feasibility of exploring the impact of epigenetic modifications on the development and maintenance of cognitive processes across the lifespan. This approach allows for the generation of data-informed hypotheses for future work aiming at characterizing and seeking to alter the molecular, cellular, and functional changes associated with the maintenance of cognition across the lifespan.

## Data Availability

The raw data supporting the conclusions of this article will be made available by the authors, without undue reservation.

## Footnotes

1
https://www.R-project.org/
